# Mortality and kidnapping estimates for the Yazidi population in the area of Mount Sinjar, Iraq, in August 2014: A retrospective household survey

**DOI:** 10.1371/journal.pmed.1002297

**Published:** 2017-05-09

**Authors:** Valeria Cetorelli, Isaac Sasson, Nazar Shabila, Gilbert Burnham

**Affiliations:** 1 Center for Humanitarian Health, Johns Hopkins Bloomberg School of Public Health, Baltimore, Maryland, United States of America; 2 Middle East Centre, London School of Economics and Political Science, London, United Kingdom; 3 Department of Sociology and Anthropology, Tel Aviv University, Tel Aviv, Israel; 4 Department of Community Medicine, College of Medicine, Hawler Medical University, Erbil, Kurdistan Region, Iraq; Massachusetts General Hospital, UNITED STATES

## Abstract

**Background:**

In August 2014, the so-called Islamic State of Iraq and Syria (ISIS) attacked the Yazidi religious minority living in the area of Mount Sinjar in Nineveh governorate, Iraq. We conducted a retrospective household survey to estimate the number and demographic profile of Yazidis killed and kidnapped.

**Methods and findings:**

The survey covered the displaced Yazidi population from Sinjar residing in camps in the Kurdistan Region of Iraq. Fieldwork took place between 4 November and 25 December, 2015. A systematic random sample of 1,300 in-camp households were interviewed about the current household composition and any killings and kidnappings of household members by ISIS. Of the 1,300 interviewed households, 988 were Yazidi from Sinjar. Yazidi households contained 6,572 living residents at the time of the survey; 43 killings and 83 kidnappings of household members were reported. We calculated the probability of being killed and kidnapped by dividing the number of reported killings and kidnappings by the number of sampled Yazidis at risk, adjusting for sampling design. To obtain the overall toll of killings and kidnappings, those probabilities were multiplied by the total Yazidi population living in Sinjar at the time of the ISIS attack, estimated at roughly 400,000 by the United Nations and Kurdish officials. The demographic profile of those killed and kidnapped was examined, distinguishing between children and adults and females and males. We estimated that 2.5% of the Yazidi population was either killed or kidnapped over the course of a few days in August 2014, amounting to 9,900 (95% CI 7,000–13,900) people in total. An estimated 3,100 (95% CI 2,100–4,400) Yazidis were killed, with nearly half of them executed—either shot, beheaded, or burned alive—while the rest died on Mount Sinjar from starvation, dehydration, or injuries during the ISIS siege. The estimated number kidnapped is 6,800 (95% CI 4,200–10,800). Escapees recounted the abuses they had suffered, including forced religious conversion, torture, and sex slavery. Over one-third of those reported kidnapped were still missing at the time of the survey. All Yazidis were targeted regardless of age and sex, but children were disproportionately affected. They were as likely as adults to be executed but constituted 93.0% (95% CI 71.9–98.6) of those who died on Mount Sinjar. Moreover, children only accounted for 18.8% (95% CI 8.4–36.9) of those who managed to escape captivity. A sensitivity analysis suggests that the actual toll of killings and kidnappings may be underestimated in our data because of survival bias. The uncertainty associated with inference from a small sample of in-camp households and the reliance on a rough figure of 400,000 for extrapolation to the total Yazidi population of Sinjar at the time of the ISIS attack are the main limitations of this study.

**Conclusions:**

Consistent with other existing evidence, our data provide a clear indication of the severity of the ISIS attack against the Yazidis in terms of both the number and demographic profile of those targeted.

## Abstract 2

Valeria Cetorelli and colleagues document the extent of the ISIS-led killings and kidnappings of the Yazidi population in 2014.

## Author summary

**Why was this study done?:**

- According to lists compiled by local authorities and human rights organisations, between 2,000 and 5,500 Yazidis were killed and more than 6,000 were kidnapped during the ISIS attack on Mount Sinjar in August 2014.
- The United Nations has not yet been able to independently verify these figures.
- This retrospective household survey provides the first population-based estimates of the number and demographic profile of Yazidis killed and kidnapped by ISIS.

**What did the researchers do and find?:**

- Between 4 November and 25 December, 2015, we collected data from a random sample of displaced Yazidi households from Sinjar living in camps in the Kurdistan Region of Iraq.
- We used these data to estimate the overall number of killings and kidnappings and the respective proportions of children and adults and females and males.
- Our estimated toll of killings and kidnappings corroborate the figures reported by local authorities and human rights organisations.
- We found that executions were indiscriminate, but children accounted for nearly all those who died on Mount Sinjar from lack of water and food or injuries during the ISIS siege; kidnappings were also indiscriminate, but children were much less likely to escape captivity than adults.

**What do these findings mean?:**

- Population-based estimates from this survey contribute to documenting the scale of the ISIS attack against the Yazidis of Sinjar.
- Combined with other existing evidence, these estimates can support a formal genocide investigation by an appointed judicial authority.

## Introduction

During the summer of 2014, the so-called Islamic State of Iraq and Syria (ISIS) subjugated Nineveh governorate in Northern Iraq. Nineveh has historically been home to most of Iraq’s minority groups, including Yazidis, Assyrian and Chaldean Christians, Sabaean-Mandaeans, Turkmen, Shabak, and Kaka’i. These minorities were systematically targeted by ISIS in its violent campaign to “purify” the region from non-Islamic influences [1]. Yazidis, whom ISIS militants consider “devil worshippers,” were singled out for particularly brutal treatment [2].

**Fig 1:**
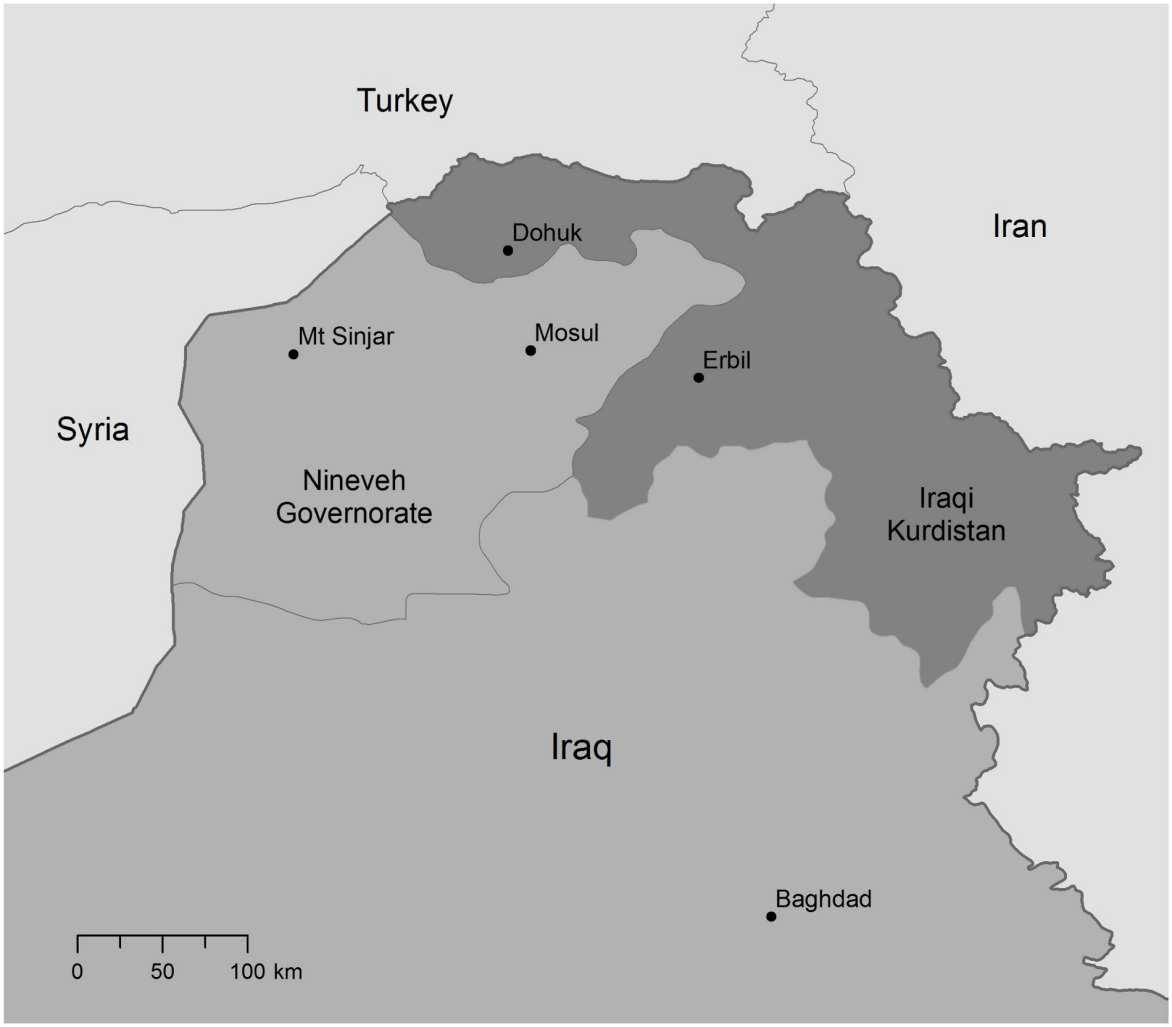
Map of Northern Iraq. Source: Authors’ adaptation of Global Administrative Areas 2.8 (www.gadm.org) and the United States National Imagery and Mapping Agency data (www.nga.mil).

The Yazidis practice an ancient religion that contains elements of Zoroastrianism, Judaism, Christianity, and Islam. They number less than 1.5 million, living mainly in Iraq, Syria, Turkey, and Armenia. The largest Yazidi community—approximately 400,000 people—resided in the area of Mount Sinjar, some 150 km west of Mosul in Nineveh governorate (Fig 1) [3,4]. The Yazidis of Sinjar have long been one of the most vulnerable and impoverished communities in Iraq. After having suffered from decades of discrimination, marginalisation, and neglect during Saddam Hussein’s regime, in recent years they have experienced increasing persecution by Sunni extremists [5].

On 3 August 2014, ISIS launched a coordinated attack on Sinjar City and surrounding towns and villages, forcing Yazidis to seek refuge on Mount Sinjar. Those who could not flee in time were either killed or kidnapped [6]. As ISIS encircled the mountain on 4 August, tens of thousands of Yazidis remained trapped without water, food, or shelter in temperatures rising above 50°C. At the request of the Iraqi government, United States airstrikes and humanitarian aid airdrops began on 8 August; helicopter rescue missions were initiated a few days later. Most Yazidis were evacuated between 9–13 August when a safe corridor was opened by Kurdish forces, allowing them to flee through Syria into the Kurdistan Region of Iraq [7].

The vast majority of Yazidis reached Kurdistan by mid-August 2014, where they found temporary shelter in hundreds of informal settlements, including schools, community spaces, and unfinished buildings. As schools were needed for the beginning of the academic year and some property owners requested the government to clear their properties, displaced Yazidis were gradually transferred to newly built camps [8]. More than 300,000 Yazidis have settled in Kurdistan, with over half in camps managed by the Kurdistan regional government and the balance still scattered in construction sites and unofficial tented settlements [8,9]. A relatively low number of Yazidis have settled elsewhere: by 2015, it was estimated that some 10,000 remained in tents on the north side of Mount Sinjar under Kurdish control, nearly 15,000 were reportedly in refugee camps in Syria, and at least 30,000 were known to have crossed into Turkey [10–12].

Soon after the events of August 2014, the United Nations Human Rights Council dispatched a mission to Kurdistan to document the scope of ISIS violations of international human rights law. The mission report asserts that “members of ISIS may have perpetrated genocide against the Yazidi community by killing, causing serious bodily or mental harm and forcibly transferring members of the group, including children, in the context of a manifest pattern of conduct aimed at the destruction of the group” and that “further investigation is needed to establish the precise number of those who continue to be held by ISIS as well as the numbers killed, estimated to be in the thousands” [13].

Some attempts have been made to compile lists of those affected. Kurdish authorities and human rights organisations have reported to the United Nations that between 2,000 and 5,500 Yazidis were killed and more than 6,000 were kidnapped; however, the United Nations has not yet been able to independently verify these figures [14]. Between 4 November and 25 December 2015, we conducted a retrospective household survey to provide the first population-based estimates of the number and demographic profile of Yazidis killed and kidnapped by ISIS.

## Methods

### Ethical approval

The study received ethical approval from the Middle East Centre Committee of the London School of Economics and Political Science, London, United Kingdom and the Ethics Committee of the Hawler Medical University, Erbil, Kurdistan Region of Iraq. Permission to conduct the survey was granted by the Kurdish Board of Relief and Humanitarian Affairs, Dohuk, Kurdistan Region of Iraq. Analysis of the data was declared exempt by the Institutional Review Board of the Johns Hopkins Bloomberg School of Public Health, Baltimore, Maryland, US.

### Data collection

The survey covered the displaced Yazidi population from Sinjar residing in camps in the Kurdistan Region of Iraq. As of December 2015, the Kurdish Board of Relief and Humanitarian Affairs (BRHA) managed 13 camps—Bajed Kandala, Bardarash, Bersive, Chamisku, Dawdiya, Essian, Garmawa, Karbato, Khanke, Mamilian, Rwanga, Shariya, and Sheikhan—hosting about 200,000 internally displaced persons (IDPs) from Nineveh governorate, predominantly Yazidis. Maps were available for all camps. All shelters (tents or prefabricated caravans) within camps were numbered.

We selected a stratified systematic random sample of 100 households per camp, yielding a total of 1,300 households. For each camp (stratum), we determined a sampling interval *k* as the ratio of camp size to sample size. We chose a random number from 1 to *k* to identify a starting household and selected every *k*th household thereafter. We defined a household as a group of people living together in one shelter. By this definition, a household corresponded in most cases to a nuclear family, but if multiple nuclear families were living in one shelter, they were still regarded as one household. In all camps, nuclear families with more than six members were given two adjacent tents or caravans; these nuclear families were regarded as one household, even though they occupied two shelters.

The questionnaire was designed to gather information about the current household composition and the age and sex of any household member who was killed or kidnapped by ISIS. Similar retrospective questionnaires have been used extensively to collect data on violence and mortality in conflict-affected populations [15–22]. To avoid double counting, respondents were asked to only report persons killed and kidnapped from their household (i.e., from the nuclear family or families living in that shelter). The relation to respondent for each reported household member was recorded. If killings of household members were reported, the circumstances of death were queried. Respondents were asked to specify whether the deceased was executed or died on Mount Sinjar during the ISIS siege. If kidnappings were reported, respondents were asked whether the person had escaped ISIS captivity or was still missing at the time of the survey. The health status of surviving household members was also queried (the health section of the survey will be published elsewhere).

The questionnaire was first drafted in English, then translated to Arabic, and then translated back to English. A focus group was organised at the Hawler Medical University to discuss major issues in content, translation, and interview flow. We revised the questionnaire based on the focus group discussion and agreed upon a consensus translation. This version of the questionnaire was pilot tested in 100 households in Shariya camp a few days before starting the survey, and final changes were made based on data evaluation and feedback from interviewers.

The field team consisted of four pairs of local Yazidi interviewers (one male and one female) and one survey supervisor. Before the pilot, the interviewers were given a three-day training session concerning the questionnaire, sampling methods, data collection using tablets, interview techniques, and basic protection principles for human subjects.

Fieldwork took place between 4 November and 25 December 2015. Interviews were conducted with the household head or a responsible adult in the household. The interviewers obtained verbal informed consent from all participants after reading a consent form outlining the purpose of the survey, its confidentiality, and the voluntary nature of participation. If no suitable informant was present in the household, an attempt was made to revisit the household later on the same day. In case of noncontact or refusal to participate, the interviewers were instructed to conduct an interview with the household living in the nearest tent or caravan. To protect the anonymity of respondents, no unique identifiers were recorded. Data were collected on tablets using Magpi 2.0. The survey supervisor was responsible for checking questionnaires for completion and quality before transmitting records from tablets to the Magpi web account.

### Statistical analysis

Data analysis was performed using Stata 14 and R 3.2, applying standard adjustment methods for unequal probability stratified sampling and a finite population correction factor [23]. Inverse probability weights were used to account for the fact that a sample of the same size was drawn from each camp but camp sizes varied from 900 to 6,000 shelters. Households occupying two shelters (i.e., those having more than six members) were reweighted to adjust for the fact that they had a higher probability of being selected into the survey.

We estimated the probability of being killed or kidnapped by dividing the number of reports by the number of sampled Yazidis at risk (i.e., excluding those born after the events on Mount Sinjar). We did not attempt to estimate the daily rate of killings and kidnappings because the exact timing of events as well as how many Yazidis were at risk at any given moment is unknown in sufficient detail. To obtain the overall toll of killings and kidnappings, those probabilities were multiplied by the total Yazidi population living in Sinjar at the time of the ISIS attack, estimated at roughly 400,000 by the United Nations and Kurdish officials [3,4]. The demographic profile of those killed and kidnapped was examined, distinguishing between children and adults and females and males. Consistent with previous surveys in Iraq, we defined children as those under 15 y of age [19–22].

Our estimates are based on the assumption that Yazidis residing in BRHA camps are representative of all Yazidis from Sinjar in terms of their exposure to the ISIS attack. We believe this assumption is reasonable given the somewhat random process through which some families were transferred from informal settlements (i.e., schools and unfinished buildings that needed to be vacated) to official camps [8]. There is no indication that in-camp and out-of-camp Yazidis are different in their exposure to the events of August 2014. On the contrary, the United Nations has provided evidence that the ISIS attack was unexpected and all Yazidis living in Sinjar were targeted [3]. The uncertainty associated with inference from a sample of in-camp households must nevertheless be acknowledged. Without a sampling frame, those Yazidis who were still scattered in informal settlements could not be included in the survey. The relatively low proportion of Yazidis who have settled elsewhere than in Kurdistan could not be included either.

Survival bias is a source of concern in this context, as there is evidence that killings and kidnappings were clustered within nuclear families. This is because those families who could not flee in time were generally captured together. Since nuclear families with no survivors had zero probability of being selected and reported in the survey, the number of killings and kidnappings is likely underestimated in our data. How large the downward bias is depends on the extent to which killings and kidnappings were clustered within families as well as the distribution of family sizes in the Yazidi population. The greater the number of families captured together and the larger those families are, the greater the underestimate.

We conducted a sensitivity analysis to assess what the underestimate of the total number of killings and kidnappings would be depending on the proportion of zero-survivor families in the Yazidi population. We simulated 10,000 random samples from a mixture distribution of family size (n_i_) and proportion of missing family members (p_i_), drawing the number of killings and kidnappings in each *i*^th^ family from a Binomial(n_i_, p_i_) distribution. The distribution of family sizes was obtained by bootstrapping, whereas the family-specific proportion of killings and kidnappings was drawn from a Beta(α, β) distribution, where α and β were fitted to the data using the method of moments. This model accounts for the greater variability (overdispersion) in our data, relative to a standard binomial model, because of the clustering of killings and kidnappings within families [24]. We obtained a sampling distribution of the overall proportion of killings and kidnappings by summing those events in each pseudosample and dividing the total by the number of individuals at risk. We used the number of nuclear families captured together in which at least one member escaped as a reference group, and we simulated what the toll of killings and kidnappings would be if a similar number of families were still in captivity or had died in their entirety with no one surviving to report.

## Results

### Sample characteristics

Of the 1,300 interviewed households in BRHA camps, 988 were Yazidi from Sinjar and 19 were Yazidi from the Nineveh Plain. The rest of the sample consisted of 105 Shabak, 6 Turkmen, 83 Sunni Kurd, 86 Sunni Arab, and 13 Assyrian Christian households. All households had been displaced from their homes in Nineveh governorate during the ISIS attack. Ninety-three of the selected households (7.2%) were replaced with households living in the nearest shelter because responsible adults were absent (6.5%) or refused to be interviewed (0.7%). The 988 Yazidi households from Sinjar contained 6,572 living residents at the time of the survey. The other 312 displaced households had 1,788 living residents (Table 1).

**Table 1:** Sample characteristics.

	Yazidis from Sinjar	Other IDPs from Nineveh
Households	988	312
Living residents	6,572	1,788
Killings Executed Died on Mt. Sinjar	43 22 21	1 1 0
Kidnappings Escaped or released Still missing	83 45 38	2 0 2

Yazidi interviewees from Sinjar reported 43 killings and 83 kidnappings of household members by ISIS. Twenty-two household members were executed—either shot, beheaded or burned alive—while 21 died from lack of water and food or injuries during the ISIS siege on Mount Sinjar. Of the 83 kidnapped, 45 had escaped ISIS captivity by the time of the survey while 38 were still missing. Killings and kidnappings included children and adults and females and males (Tables 2 and 3). Among others interviewed, one killing attributed to ISIS was reported by a Sunni Arab household from the Nineveh Plain and two kidnappings were reported by Shabak households from Mosul. In all three cases, the dead or missing person was an adult male.

**Table 2:** Reported killings and kidnappings of children (0–14) and adults (15+) in the sample.

	Yazidis from Sinjar		Other IDPs from Nineveh	
	Children	Adults	Children	Adults
Total	55	71	0	3
Killings Executed Died on Mt. Sinjar	27 8 19	16 14 2	0 0 0	1 1 0
Kidnappings Escaped or released Still missing	28 9 19	55 36 19	0 0 0	2 0 2

**Table 3:** Reported killings and kidnappings of females and males (all ages) in the sample.

	Yazidis from Sinjar		Other IDPs from Nineveh	
	Females	Males	Females	Males
Total	68	58	0	3
Killings Executed Died on Mt. Sinjar	23 10 13	20 12 8	0 0 0	1 1 0
Kidnappings Escaped or released Still missing	45 27 18	38 18 20	0 0 0	2 0 2

### Population estimates

**Fig 2:**
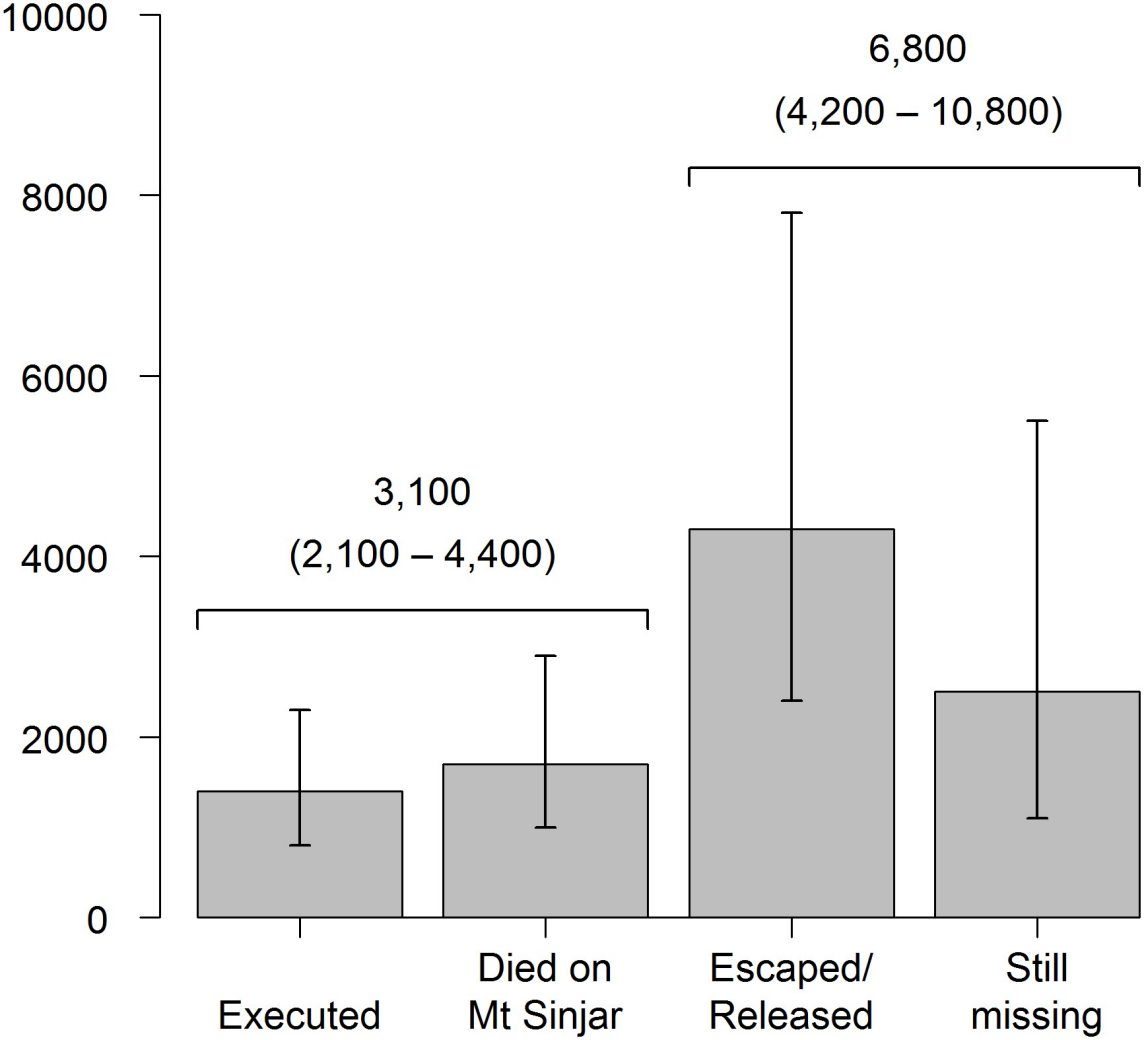
Estimated toll of killings and kidnappings with 95% CIs.

Table 4 shows the estimated probabilities of killings and kidnappings of Yazidis by ISIS. Nearly 25 out of 1,000 people were either killed or kidnapped (95% CI 17.5–34.6). The likelihood of being killed was 7.6 per 1,000 (95% CI 5.3–11.0) and the likelihood of being kidnapped 17.0 per 1,000 (95% CI 10.7–26.9). Fig 2 displays the estimated toll of killings and kidnappings, assuming a Yazidi population of 400,000 in Sinjar at the time of the ISIS attack. The overall estimated toll is 9,900 (95% CI 7,000–13,900) people. An estimated 3,100 (95% CI 2,100–4,400) Yazidis were killed: 1,400 (95% CI 800–2,300) were executed, and 1,700 (95% CI 1,000–2,900) died on Mount Sinjar during the ISIS siege. The estimated number kidnapped is 6,800 (95% CI 4,200–10,800). At the time of the survey, an estimated 4,300 (95% CI 2,400–7,800) Yazidis had escaped captivity, while an estimated 2,500 (95% CI 1,100–5,500) were still missing.

**Table 4:** Estimated probabilities of killings and kidnappings of Yazidis from Sinjar.

	Events per 1,000 people (95% CIs)
Total	24.6 (17.5–34.6)
Killings Executed Died on Mt. Sinjar	7.6 (5.3–11.0) 3.4 (2.1–5.6) 4.2 (2.4–7.2)
Kidnappings Escaped or released Still missing	17.0 (10.7–26.9) 10.9 (6.1–19.3) 6.1 (2.7–13.6)

Killings and kidnappings were highly clustered—6.7% of nuclear families in the sample accounted for all these events. Of those nuclear families with at least one killing or kidnapping, 13.2% had all members either killed or kidnapped but were reported in the sample because at least one family member managed to escape ISIS captivity. In other words, nearly 1% of the nuclear families in our sample were captured in their entirety. Had there been no one to escape and report their fate, our estimate of the total number of killings and kidnappings would have been 21.7% lower, suggesting that the potential underestimate is large.

**Fig 3:**
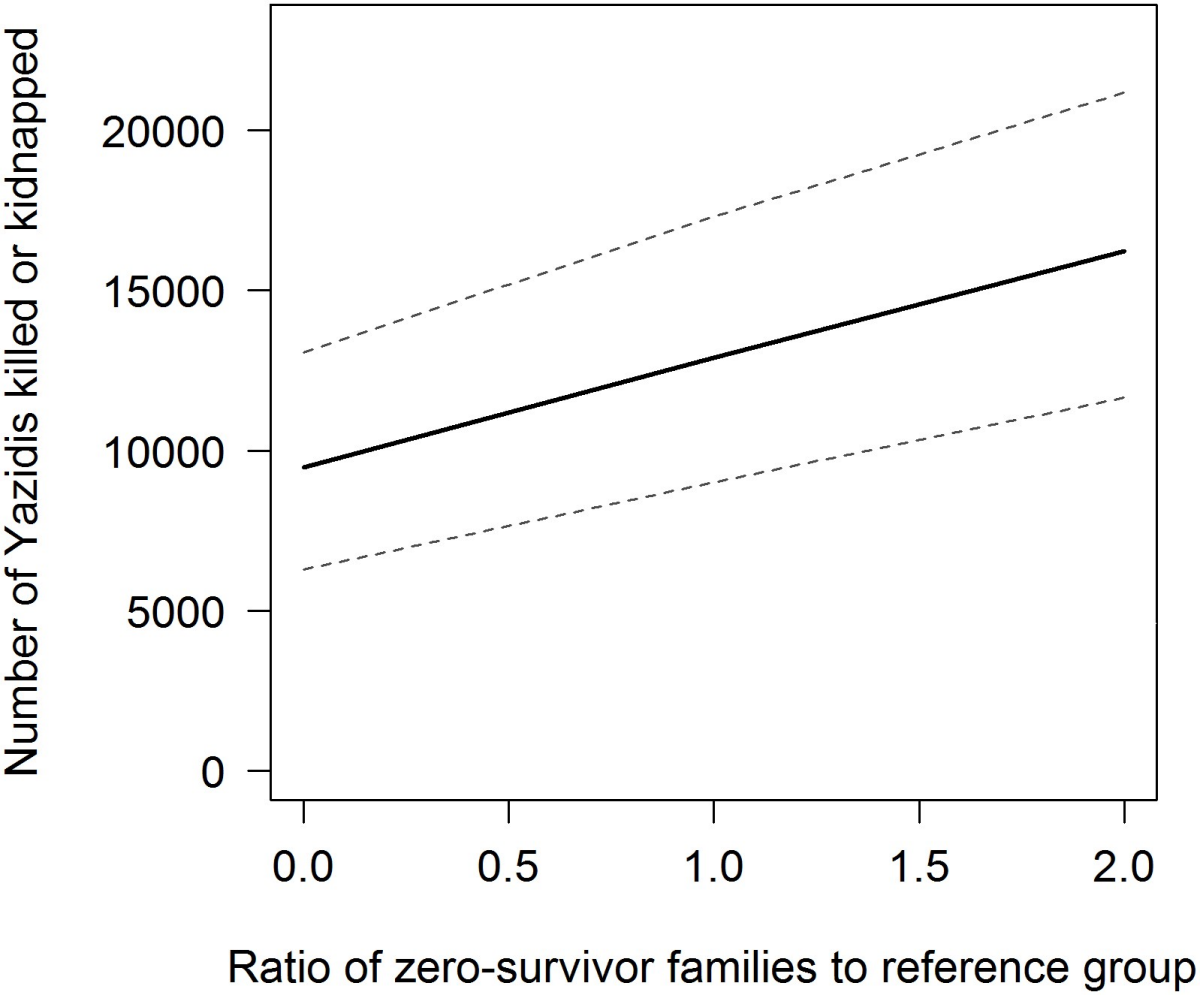
Sensitivity analysis to number of zero-survivor families with 95% CIs. Note: reference group = nuclear families captured in their entirety in which at least one member escaped.

Fig 3 shows the sensitivity of our estimate to the potential number of zero-survivor families. The proportion of nuclear families in the sample who were captured in their entirety but subsequently had one or more members escape provides a useful point of reference. If an equal number of nuclear families are still missing, the toll of killings and kidnappings would be 12,900 (95% CI 9,000–17,300). Although the expected toll of killings and kidnappings increases linearly as a function of the number of zero-survivor families, as expected, the confidence band surrounding that number tends to widen. Contributing to the rising uncertainty are the clustering of killings and kidnappings within families and, to a lesser extent, the variation in family size in the population.

**Fig 4:**
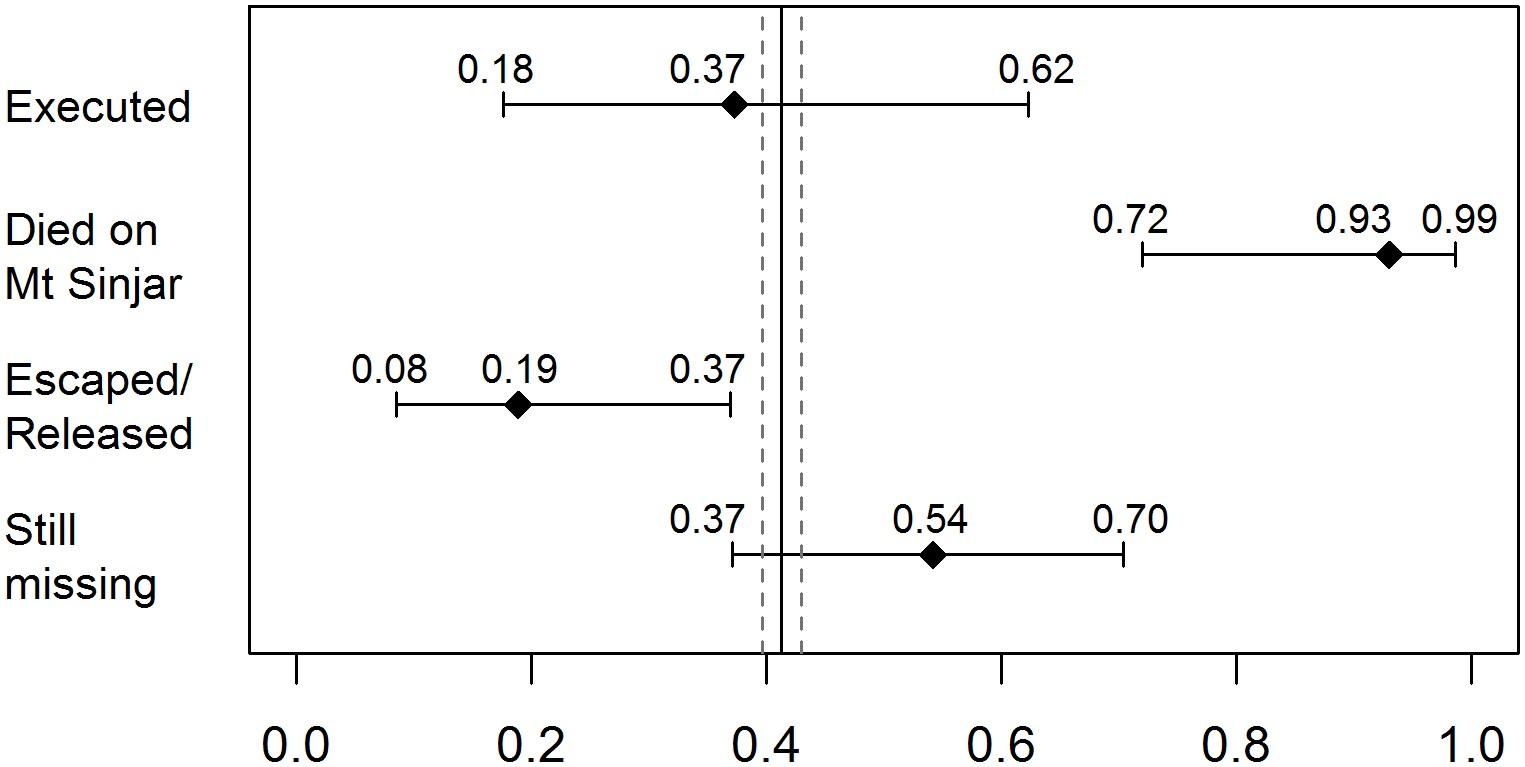
Estimated proportion of children (0–14) among all killings and kidnappings with 95% CIs. Note: The vertical line is the estimated proportion of children (0–14) in the Yazidi population with 95% CI.

**Fig 5:**
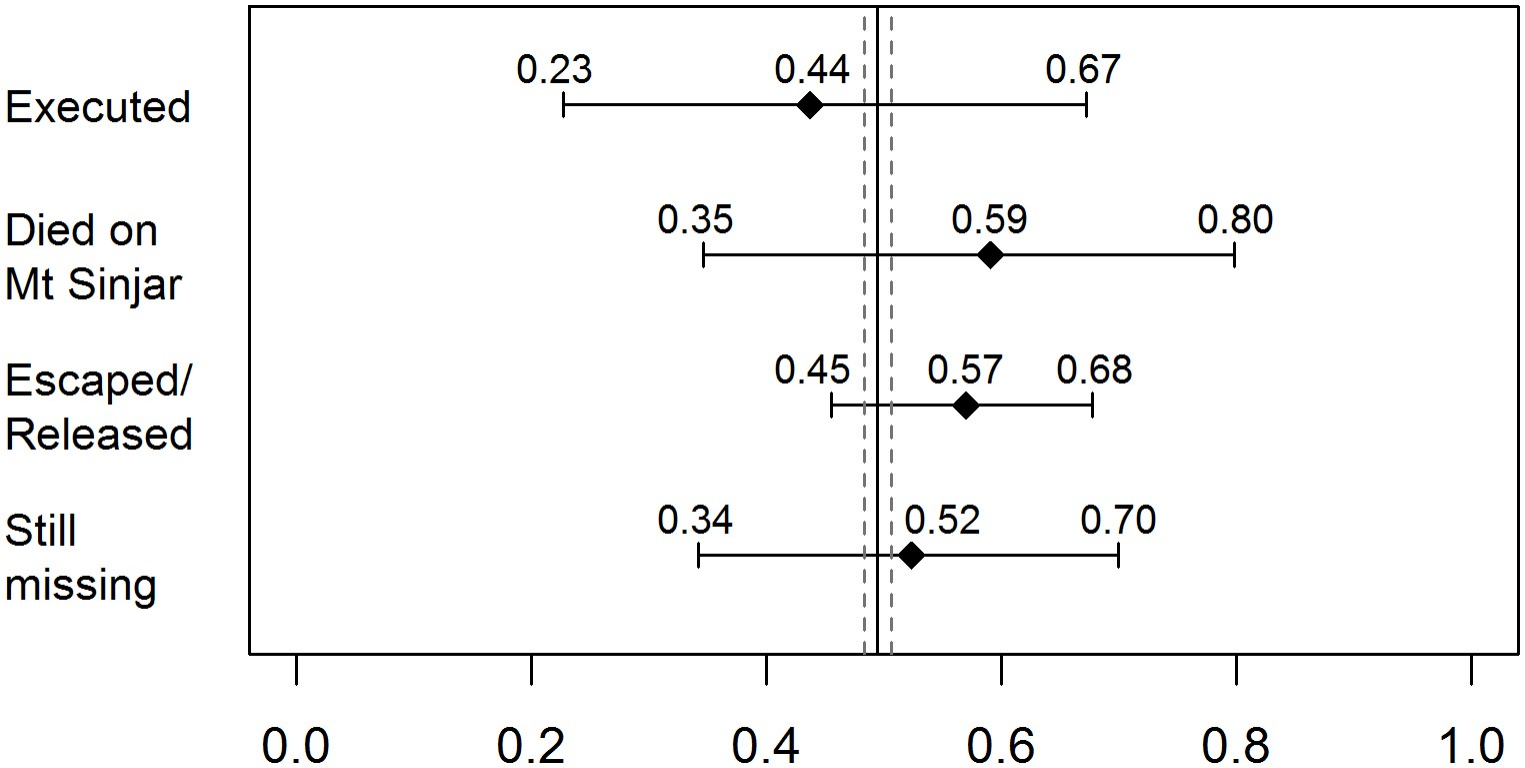
Estimated proportion of females (all ages) among all killings and kidnappings with 95% CIs. Note: The vertical line is the estimated proportion of females (all ages) in the Yazidi population with 95% CI.

Figs 4 and 5 outline the age and sex profile of Yazidis killed and kidnapped. At the time of the ISIS attack, the proportion of children under 15 y within the population was 41.2% (95% CI 39.6–42.9), and the proportion of females of all ages was 49.4% (95% CI 48.3–50.6). Children were disproportionately affected by ISIS violence. They were as likely as adults to be executed (95% CI 17.6–62.3) but constituted 93.0% (95% CI 71.9–98.6) of those who died on Mount Sinjar. Moreover, children only accounted for 18.8% (95% CI 8.4–36.9) of those who managed to escape captivity. Females were as likely as males to be executed (95% CI 22.7–67.2) and to die on Mount Sinjar (95% CI 34.6–79.8). They were also as likely to be kidnapped and still missing (95% CI 34.2–69.9).

## Discussion

The Yazidi religious minority of Sinjar was devastated by the ISIS attack of August 2014. We estimate that 2.5% of the Yazidi population was either killed or kidnapped over the course of just a few days. Other minority groups in Nineveh governorate were also attacked on the basis of their religious identities and forced to flee their homes, but the scale of killings and kidnappings was not as high [2,3]. The Yazidis have long faced discrimination in Iraq and in recent years have experienced increasing persecution by Sunni extremists. In 2007, two Yazidi villages were completely destroyed in the single most devastating Islamist terror attack since the beginning of sectarian conflict in the country [5].

While violent deaths in Iraq have been typically concentrated among adult men [19–22,25], our data show that Yazidis were targeted by ISIS regardless of age and sex. Executions were indiscriminate, including children and adults and females and males. This finding is confirmed by the discoveries of 35 mass graves containing the remains of hundreds of Yazidi men, women, and children in the Sinjar area that has been taken back from ISIS [26]. Our data show that nearly all of those who died on Mount Sinjar from starvation, dehydration, or injuries during the ISIS siege were children. This finding is consistent with a UNICEF statement on 5 August 2014 expressing extreme concern for up to 25,000 children stranded on the mountain “in dire need of humanitarian aid including drinking water and sanitation services” [27]. Similar warnings appeared in contemporary news reports [28].

Kidnappings were also indiscriminate. Families in captivity were separated and forcibly transferred to different locations in ISIS-controlled territory. Escapees recounted the abuses they had suffered, including forced religious conversion, torture, and sex slavery. The outcomes of kidnapping varied. Children under 15 y of age were much less likely to escape captivity than adults. Several accounts confirm that girls were sold or gifted to ISIS fighters, while boys were forced to attend ISIS training camps [3,29–31]. It is currently impossible to establish how many of those still missing are alive.

We estimate that 3,100 (95% CI 2,100–4,400) Yazidis were killed and 6,800 (95% CI 4,200–10,800) were kidnapped. These figures are aligned with the most recent counts reported to the United Nations by Kurdish authorities and human rights organisations (i.e., between 2,000 and 5,500 killings and more than 6,000 kidnappings) [14]. The fact that multiple independent sources arrived at similar estimates increases our confidence in the general findings [32].

Nevertheless, this survey has some limitations that must be acknowledged. First, our estimates are based on a random sample of Yazidi households residing in BRHA camps, assuming that their exposure to the ISIS attack is representative of the experience of the whole Yazidi population from Sinjar. This assumption seems reasonable given the unexpected and indiscriminate nature of the attack and the somewhat random process thought which some of the displaced Yazidis were transferred from informal settlements to official camps [3,8]. However, the possibility of a higher or lower proportion of killings and kidnappings among Yazidis who are still scattered in informal settlements in Kurdistan or among those who have settled elsewhere cannot be completely ruled out, and a survey of out-of-camp households cannot be conducted under the present circumstances. Further uncertainty in our estimates is introduced by the reliance on a rough figure of 400,000 for extrapolation to the Yazidi population of Sinjar at the time of the attack, as the exact number is unknown [3,4].

We believe that the actual toll of killings and kidnappings may be underestimated in our data because of survival bias. A retrospective survey requires at least one surviving member to report killings and kidnappings in the family; hence, it is precisely the families with the largest number of members killed or still in captivity which are excluded from our sample. Although the number of zero-survivor families is unknown, it is possible that it is in the vicinity of the number of families captured together who later escaped, whether in whole or in part. If this is the case, the overall toll of killings and kidnappings would be higher than our estimate by several thousands. New measurement methods need to be developed to address the problem of survival bias in crisis settings, particularly in contexts where families tend to be targeted as a whole.

## Conclusion

On the occasion of the second anniversary of the August 2014 attack, the United Nations Human Rights Council has declared that the ISIS violence against Yazidis constitutes a case of ongoing genocide, calling for a refocus of attention on the rescue, protection, and care of the Yazidi community and recommending the Security Council to refer the case to the International Criminal Court or an ad hoc tribunal with relevant geographical and temporal jurisdiction [33]. The Convention on the Prevention and Punishment of the Crime of Genocide defines genocide as “any of the following acts committed with intent to destroy, in whole or in part, a national, ethnical, racial or religious group, as such: (a) killing members of the group; (b) causing serious bodily or mental harm to members of the group; (c) deliberately inflicting on the group conditions of life calculated to bring about its physical destruction in whole or in part; (d) imposing measures intended to prevent births within the group; (e) forcibly transferring children of the group to another group” [34]. A United Nations Commission of Inquiry has recently determined that “ISIS has committed, and is committing, the prohibited acts with the intent to destroy, in whole or in part, the Yazidis of Sinjar” [3]. Population-based estimates from our survey confirm the severity of the ISIS attack against the Yazidis. Combined with other existing evidence, these estimates can contribute to documenting the full scale of violations and holding perpetrators accountable for their actions.

## Supporting information

S1 Questionnaire (PDF)

S1 STROBE statement (PDF)

## Abbreviations

BRHA: Board of Relief and Humanitarian Affairs
IDP: internally displaced person
ISIS: Islamic State of Iraq and Syria
