# Supplementary material for: Mortality and kidnapping estimates for the Yazidi population in the area of Mount Sinjar, Iraq, in August 2014: A retrospective household survey
Source: PLoS Med. 2017 May 9;14(5):e1002297. doi: 10.1371/journal.pmed.1002297 (PMC5423550; doi:10.1371/journal.pmed.1002297)
Supplement: S1 Questionnaire — (PDF) [file pmed.1002297.s001.pdf]

Verbal consent form

Hello, my name is ________. I am part of a team from Hawler Medical University conducting a survey of households in ________ camp to understand events that you and those in your household experienced during displacement. We will also ask some questions about your health and the health of those in your household. The interview will take about 20 minutes.

The information you provide will help improve the health services as well as improve the protection of persons who must flee their homes for safety in times of war.

We will not write down your full name, and everything that you tell us will be kept completely confidential. No one can find out what you tell us. We would like to ask you these questions in private so no one can overhear your answers.

Taking part in this survey is completely voluntary. You don't have to answer questions, and even if you start you can change your mind and stop at any time. If you decide not to participate, you can still receive the same health and other services you have received in the past. You will not receive any rewards for participating.

May I start asking you questions now?

________ *interviewer initials indicating consent to participate.*

نموذج الموافقة اللفظية

مرحبا، انا اسمي ________. أنا جزء من فريق جامعة هولير الطبية الذي يقوم بإجراء مسح للأسر في مخيم ________ لفهم الأحداث التي واجهتها انت وأعضاء أسرتك أثناء النزوح. سنسألك بعض الأسئلة حول صحتك وصحة افراد أسرتك. سوف تستمر المقابلة حوالي 20 دقيقة.

المعلومات التي ستقدمها تساعد على تحسين الخدمات الصحية فضلا عن تحسين حماية الأشخاص الذين يفرون من بيوتهم للسلامة في أوقات الحرب.

نحن لن نكتب اسمك الكامل، وكل ما تقوله لنا سيبقى سري تماما. لا أحد يستطيع معرفة ما تخبرنا به. نود أن نسألك هذه الأسئلة على الانفراد لكي لا يتمكن أحد من سماع إجاباتك.

المشاركة في هذه الدراسة هو طوعي تماما. لا يوجب عليك الإجابة على الأسئلة، وحتى لو بدأت يمكنك أن تغير رأيك والتوقف في أي وقت. إذا قررت عدم المشاركة، سيظل بإمكانك الحصول على نفس الخدمات الصحية وغيرها التي كنت تحصل عليها في الماضي. سوف لن تتلقى أي مكافأة عن المشاركة.

هل استطيع ان ابدأ بطرح الأسئلة الآن؟

________ *الاسم الاول للشخص الذي يجري المقابلة للاشارة بالموافقة على المشاركة.*

Main Form

النموذج الرئيسي

Date of interview

تأريخ المقابلة

Camp number

رقم المخيم

Household number

رقم الأسرة

Interviewer name

أسم الشخص الذي أجرى المقابلة

Did the respondent provide consent for this interview?

هل ابدى المجيب الموافقة على اجراء هذه المقابلة؟

Would you please list all the adults and children who live in this household including yourself?
(A household is a group of people living together in the selected shelter)

يرجى ادراج جميع الاشخاص البالغين و الاطفال الذين يعيشون في هذه الاسرة بما في ذلك نفسك
(الأسرة هي مجموعة من الناس يعيشون معا في المأوى المختار)

Household List Sub-Form

النموذج الفرعي لقائمة الأسرة

Household member number

رقم عضو الأسرة

First name or initial of household member

الأسم الأول لعضو الأسرة

Age at last birthday (if under 1 year, enter "0.months")

العمر في اخر عيد ميلاد (اذا اقل من سنة، ادخل "0.الاشهر")

Sex

الجنس

Highest level of education completed

أعلى مستوى دراسي تم اكماله

Relation to respondent

العلاقة بالمجيب

End of sub-form

نهاية النموذج الفرعي

Is this correct that there are ____ persons in this household?

هل صحيح انه يوجد ____ شخص في هذه الأسرة؟

What is the religion of this household?

ما هي ديانة هذه الاسرة؟

Where was the household's permanent residence before displacement? (governorate and district)

أين عاشت هذه الأسرة بشكل دائم قبل التهجير؟ (المحافظة و القضاء)

When did you arrive to settle here? (month and year)

متى وصلت لتستقر هنا؟ (الشهر و السنة)

Did any member of this household die while fleeing?

هل توفي اي من اعضاء هذه الأسرة عند الهروب؟

Please enter data about death of any member of this household

الرجاء ادخال البيانات حول وفاة اي عضو من هذه الأسرة

Deaths Sub-Form

النموذج الفرعي للوفاة

Please enter the person number (following the household listing) of the deceased household member

يرجى ادخال رقم الشخص (حسب قائمة الأسرة) لعضو الأسرة المتوفي

First name or initial of household member

الاسم الاول لعضو الاسرة

Sex

الجنس

Date of death (month and year)

تأريخ الوفاة (الشهر و السنة)

Age at death (if under 1 year, enter "0.months")

العمر عند الوفاة (اذا اقل من سنة، ادخل "0.الاشهر")

Relation to respondent

العلاقة بالمجيب

Cause of death

سبب الوفاة

End of sub-form

نهاية النموذج الفرعي

Was any member of this household kidnapped?

هل تم اختطاف اي عضو من هذه الأسرة

Please enter data about kidnapping of any member of this household

يرجى ادخال البيانات حول اختطاف اي عضو من هذه الاسرة

Kidnappings Sub-Form

النموذج الفرعي للاختطاف

Please enter the person number (following the household listing) of the kidnapped household member

يرجى ادخال رقم الشخص (حسب قائمة الأسرة) لعضو الأسرة المخطوف

First name or initial of household member

الأسم الأول لعضو الأسرة

Sex

الجنس

Date of kidnapping (month and year)

تأريخ الاختطاف (الشهر و السنة)

Age at kidnapping (if under 1 year, enter "0.months")

العمر عند الأختطاف (اذا اقل من سنة، ادخل "0.الاشهر")

Relation to respondent

العلاقة بالمجيب

Current status of the kidnapped household member

الوضع الحالي لعضو الأسرة المخطوف

End of sub-form

نهاية النموذج الفرعي

INTERVIEWER: This is the end of the questionnaire. Please thank the participant for their time and write any additional comment you might have.

الشخص الذي يجري المقابلة: هذا نهاية الاستبيان. يرجى شكر المشارك لوقتهم و اكتب اية ملاحظة اضافية قد تكون لديك.
